# Bonding Performance of a Hydrophilic Amide Monomer Containing Adhesive to Occlusal and Cervical Dentin

**DOI:** 10.3390/ma13214727

**Published:** 2020-10-23

**Authors:** Eri Seitoku, Shuhei Hoshika, Takatsumi Ikeda, Shigeaki Abe, Toru Tanaka, Hidehiko Sano

**Affiliations:** 1 Faculty of Dental Medicine, Hokkaido University, Sapporo 060-8586, Japan; seitoku3045@den.hokudai.ac.jp (E.S.); starplatinum777@hotmail.com (S.H.); iketaka@den.hokudai.ac.jp (T.I.); tanto@den.hokudai.ac.jp (T.T.); sano@den.hokudai.ac.jp (H.S.); 2 Graduate School of Biomedical Sciences, Nagasaki University, Nagasaki 852-8102, Japan

**Keywords:** hydrophilic amide monomer, microtensile bond strength, failure modes, scanning electron microscope

## Abstract

This study aimed to evaluate the bonding performance of a new one-step self-etching adhesive system containing a novel hydrophilic amide monomer. Clearfil Universal Bond Quick (CUB) and Clearfil Megabond 2 (CMB) were used as the one-step and two-step adhesive systems, respectively. Flat dentin surfaces of human premolars were exposed using #600 SiC (silicon carbide) and bonded with the respective adhesives of each system. The teeth were sectioned to obtain beams (1 mm × 1 mm) after 24 h of water storage. The mean bond strength and standard deviations (MPa) on an occlusal surface were as follows: CUB: 45.9 ± 19.7 and CMB: 67.9 ± 25.3. The values for cervical ones were CUB: 56.0 ± 20.3 and CMB: 67.6 ± 16.0, respectively. In both conditions, the microtensile bond strength (μTBS) value was lower than that of CMB. As seen during the microscopic observation, no adhesive failure was observed after μTBS testing because CUB formed a firm and tight adhesive interface.

## 1. Introduction

In recent years, it has been recommended that restoration should be made with minimum removal of tooth structure based on the concept of “Minimal Intervention” [1,2]. From one point of view, composite resin restoration might be the most valuable method, because the restoration could be esthetic, handy and tooth conservative. In composite resin restoration, the most crucial item would be the adhesive system.

In the first stage of dental adhesives to dentin, it was performed through three steps (etching, priming and bonding). Owing to the demand for simpler as well as more user-friendly and less technique sensitive adhesives, manufacturers have developed new adhesives used in two- or one-step procedures [3]. Regarding the one-step self-etch adhesives (1-SEAs), some researchers reported their comparable bonding performance with that of two-step self-etch adhesives (2-SEAs) [4,5,6]; however, other researchers reported their inferior performance [7,8].

A novel 1-SEA, Clearfil Universal Quick (Kuraray Noritake Co., Tokyo, Japan), containing a newly developed hydrophilic amide monomer has become commercially available. This hydrophilic amide monomer would have a higher setting ability; consequently, the formed polymer would be resistant to hydrolysis. Furthermore, as the monomer has a higher hydrophilic potential than 2-hydroxyethyl methacrylate (HEMA), it has good wettability to tooth structure [9,10]. Given the higher wettability of this monomer, the manufacturer insists that immediate air-drying after application of the adhesive should be performed. If done, it could be advantageous for good adhesion, especially in the cervical region, because the shorter manipulation time could avoid irritation from adhesion-reducing factors such as bleeding from the gingiva, gingival crevicular fluids, and moisture in the oral cavity, among others [11]. Furthermore, this 1-SEA can also be used for pretreatment in abutment construction using composite resin and the pretreatment of resin cements in the luting of prostheses [12]. In these cases, an adhesive interface would be formed on the cervical dentin surface, approximately perpendicular to the long axis of the tooth as the cervical force occurred [13]. If the adhesion at this region were to be broken, it would result in marginal leakage; consequently, secondary caries would occur. Hence, it is clinically crucial to form reliable adhesion at the cervical region for the successful use of universal type 1-SEA. Therefore, this study aimed to evaluate the bonding performance of the novel hydrophilic amide monomer containing 1-SEA on cervical dentin.

## 2. Materials and Methods

### 2.1. Materials Used

Basic information on the materials used herein can be found in Table 1. The 1-SEA and 2-SEA, Clearfil Universal Bond Quick (CUB) and Clearfil Megabond 2 (CMB) (Kuraray Noritake Dental Co. Tokyo, Japan), respectively, were used in this study.

### 2.2. Adhesive Specimen Preparation

**Figure 1:**
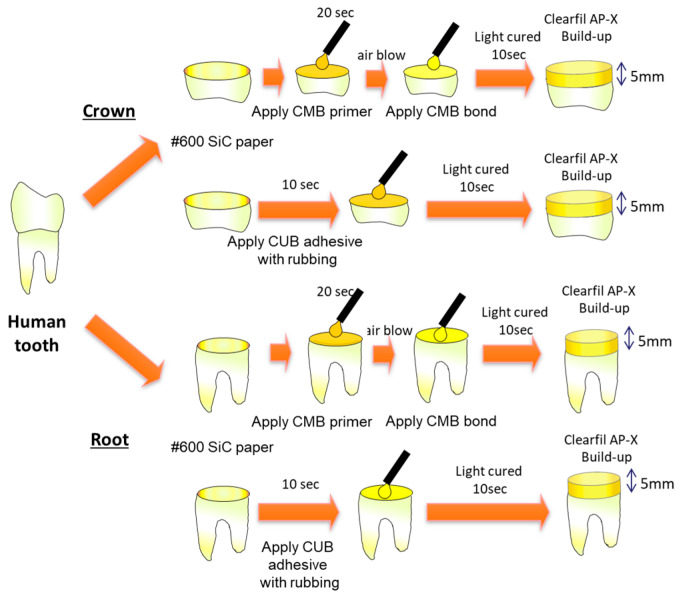
Procedure for specimen preparation.

Details regarding adhesive specimen preparation are presented in Figure 1. The collected teeth were stored following the approval of the committee of Ethics at Hokkaido University (#2013-7). Extracted teeth were stored following the guideline [14] until the experiment. The teeth were stored at 4 °C in 0.5% chloramine-T aqueous solution and used within 4 months after extraction. Each tooth was cut into crown and root. Regarding the coronal part, flat dentin surfaces were obtained by removing the coronal enamel of each tooth in a trimmer (Model Trimmer; Morita, Tokyo, Japan). Subsequently, the dentin surfaces were ground with #600 SiC (silicon carbide) paper for 60 s under water-cooling to produce a standardized smear layer prior to bonding (occlusal surface) [15]. For the root part, the cut surface was then ground with 600-grit SiC paper for 60 s. The contained occlusal and cervical surfaces were applied using CUB and CMB, as described in Figure 1 (application time, 10 s). After the application of the two adhesives, CLEARFIL AP-X (Kuraray Noritake Dental, Tokyo, Japan) were built on the surface using a light emitting diode light-curing device (Pencure 2000; Morita, Tokyo, Japan) for 40 s. The number of specimens for each of the four groups (two adherends × two adhesives) was three. The details were described in [16].

### 2.3. Microtensile Strength Testing

**Figure 2:**
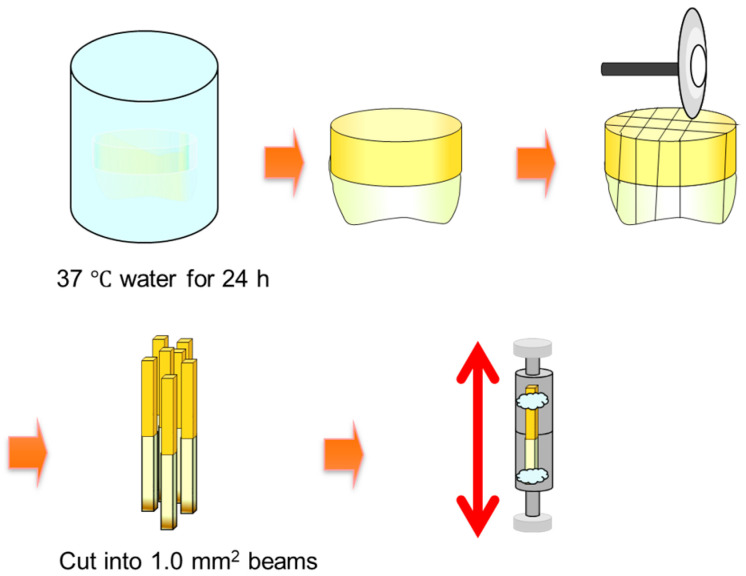
Procedure for microtensile bond strength testing.

The procedure of evaluation of μTBS is displayed in Figure 2. For both substrates, coronal and root, the dentin was prepared and bonded following the manner described in the current article. Bond strength testing was exactly following the procedure described in the previous reports [17]. The load at failure was recorded in Newtons and divided by the bonding surface area in square mm to calculate the bond strength in MPa [14,18]. Statistical analyses for the four groups were conducted using a two-way analysis of variance (ANOVA) test (*p* = 0.05)

### 2.4. Failure Mode

After the tensile test, dentin-side specimens were stored in a dry environment. Following desiccation, the failure surfaces of the specimens were observed through a digital microscope (VHX-5000; Keyence, Osaka, Japan) at a magnification of ×20 and recorded as either a “cohesive failure in composite resin,” a “mixed failure at the interface and cohesive in dentin, ” or a “cohesive failure in dentin.”

### 2.5. Scanning Electron Microscope (SEM) Observation of the Adhesive Interface

Specimens were stored in water (37 °C) for 24 h. Then, the specimens were cut in the plane perpendicular to the adhesive interfaces (1.5 mm in thickness) using a low-speed diamond saw under water cooling. Subsequently, the specimens containing the adhesive interface were polished with SiC paper under water, and then followed by diamond paste in wet conditions. The polished surface was treated with 1 mol/L phosphoric acid and 5% NaOCl aqueous solution. The surfaces of the specimens were Pt-Pd coated for SEM observation (E-1030 Ion Sputter, HITACHI, Tokyo, Japan). Coated dentin surfaces were observed with field emission scanning electron microscope (FE-SEM, S-4000: HITACH, Tokyo, Japan) at an accelerated voltage of 10kV. The details were described in [19].

## 3. Results

### 3.1. μTBS

**Figure 3:**
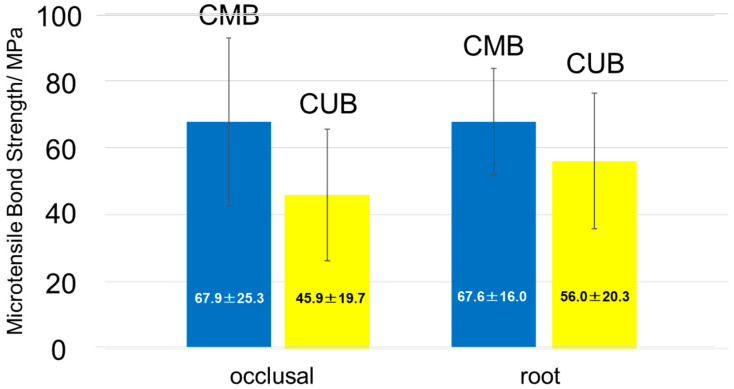
Microtensile bond strength (μTBS). CMB (blue), CUB (yellow); (*p* > 0.05).

The results of μTBS testing are shown in Figure 3. In this study, no specimen failed before μTBS testing. The μTBS of CMB on the occlusal surface (CMB−occlusal) and CUB on the occlusal surface (CUB−occlusal) were 67.9 ± 25.3 MPa (*n* = 48) and 45.9 ± 19.7 MPa (*n* = 43), respectively. Furthermore, the μTBS of CMB on the cervical surface (CMB−cervical) and CUB on the cervical surface (CUB−cervical) were 67.6 ± 16.0 MPa (*n* = 35) and 56.0 ± 20.3 MPa (*n* = 37), respectively. As a result of the two-way ANOVA, CMB showed a significantly higher μTBS than CUB irrespective of adherends (*p* < 0.05). No significant difference was observed between the two adherends (*p* > 0.05).

### 3.2. Failure Modes

**Figure 4:**
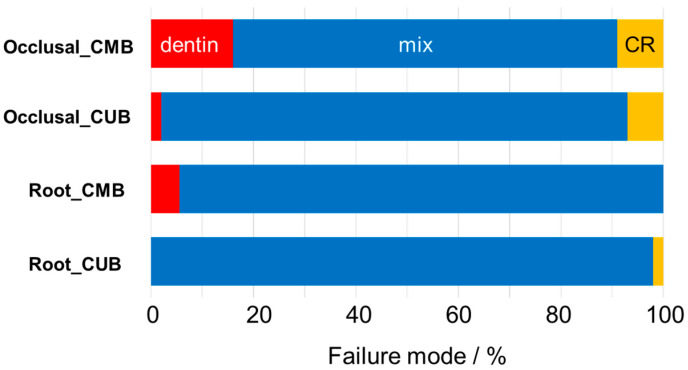
Failure modes. Cohesive failure in dentin (red), mixed failure (blue), cohesive failure in resin (yellow).

The failure modes after μTBS testing are indicated in Figure 4. For all the groups, “mixed failure at interface and cohesive in dentin” was the dominant mode. No specimen failed only at the adhesive interface in any of the groups.

### 3.3. SEM Observation of the Adhesive Interface

**Figure 5:**
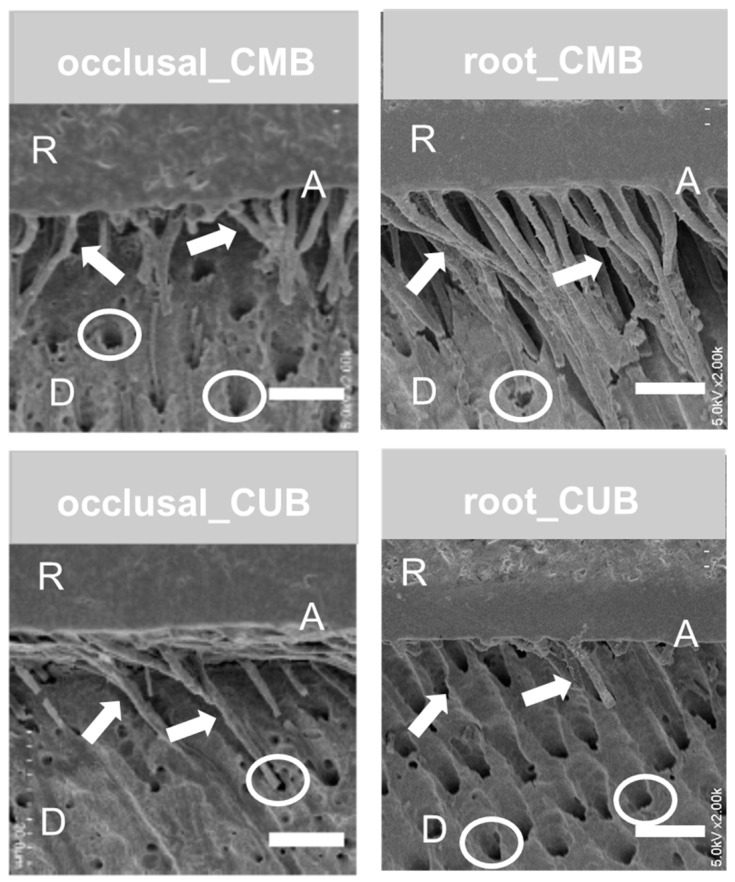
SEM images of adhesive interface. A: adhesive layer, R: composite resin, D: dentin, white arrows: resin tag, white circle: dental tubules. Scale bar: 30 μm.

Interfacial SEM images of CMB−occlusal, CUB−occlusal, CMB−cervical and CUB−cervical are shown in Figure 5. No droplet, which acts as a sign of phase-separation was observed in the adhesive layer in both CMB and CUB specimens. Furthermore, no gap formation between the adhesive and the dentin was seen in the four groups. In the cases of CMB−occlusal and CMB−cervical, numerous resin tags that were formed in dentinal tubules could be seen. Conversely, few resin tags were seen in CUB−occlusal and CUB−cervical.

## 4. Discussion

In this study, CUB indicated a lower μTBS, than that of CMB, regardless of whether adherends were cervical or occlusal (*p* < 0.05). Generally, one reason for the inferior bonding performance of 1-SEAs compared to that of 2-SEAs is a defect in the adhesive interface caused by the phase separation of the hydrophilic and the hydrophobic part contained in 1-SEAs [20]. Van Landuyt et al. revealed that some HEMA-free 1-SEA systems contained a hydrophobic monomer mixture, with which residual water could cause phase separation and blister formation in the adhesive layer [21]. The large blisters in the adhesive layer were generated by the phase separation. Consequently, the blisters acted as a weak point in the layer and adversely affected bond strength. In this study, no defect such as a “bubble” was observed in the adhesive layer of CUB. For hydrophilic properties, CUB contains hydrophilic monomers such HEMA and a hydrophilic amide monomer is contained in CUB. This hydrophilic amide monomer has a higher hydrophilicity in comparison with HEMA. As hydrophilic parts such as water were solved in the two monomers, phase separation could not occur in CUB. Thus, the defect caused by phase separation could not be a reason for the lower bond strength of CUB.

Commonly, it has been reported that 1-SEAs indicate a lower strength of adhesive than 2-SEAs. This fact can be explained by the lower degree of conversion, because 1-SEAs contain water and solvent(s) in their adhesive solution [22]. As for CUB, the polymerization properties of the hydrophilic amide monomer are considerably higher than HEMA. Therefore, the strength of the set adhesive is expected to be higher than that of conventional 1-SEAs. Kuno et al. reported that they prepared a hydrophilic 1-step self-etching adhesive which has the same composition as CUB and contained HEMA instead of hydrophilic amide monomer (denoted as CUB−H) [23]. Then they compared with ultimate tensile strength (UTS) of polymerized adhesives. The μTBS to dentin of both of them was also determined. CUB showed significantly higher UTS and μTBS than CUB−H. However, it seems difficult for the strengths of 1-SEAs to become superior to those of the bonding agents contained in 2-SEAs. Future studies should focus on the strengths of the cured adhesive of CUB in comparison to conventional 1-SEAs and the bond of 2-SEAs.

Regarding one of the major shortcomings of 1-SEAs, some researchers have reported that the bonding effectiveness of particularly (ultra-) mild self-etch adhesives might be impaired by thick smear layers [24,25].

In this study, numerous resin tags were formed in CMB−occlusal and CMB−cervical; however, few resin tags were seen in CUB−occlusal and CUB−cervical. This might be because the application time of CUB (10 s) was shorter than that of the primer of CMB (20 s). Both of the two adhesive systems used herein were (ultra-) mild self-etch adhesives. The shorter application time of CUB might have resulted in insufficient smear removal on the intertubular dentin surface and smear-plug in dentinal tubules. Hence, this fact might be one of the reasons for the lower μTBS for CUB in this study. In the present study, a smear layer was created using #600 SiC paper, although a thicker smear layer could be formed by regular diamond bars clinically. Furthermore, the application time recommended by the manufacturer is “0 (no waiting)” for CUB. Thus, many more remnants of the smear layer might be inhibited by satisfactory bonds in clinical use. Therefore, to avoid this, clinicians should ensure that a “rubbing motion” is performed, and it might be better to finish cavities and preparations using ultrafine diamond bars [25]. Future studies should focus on the influence of such factors, namely, the rubbing motion, application time and smear thickness, on the bonding performance of CUB. Moreover, the presence of defects in the tooth surface is also an important viewpoint. Grassi et al. investigated the effect of defects for endodontic sealers on bond strength of the dentin postinterface [26]. When the transverse section area of the cement layer contained defects at 12% or more, the interfacial shear strength decreased ca. 30% compared with a nondefect sample. In addition, Pettini et al. revealed that in vivo and in vitro cytogenetic and genotoxic effects by dental composite materials indicated different results [27]. Such an investigation should be required for a new material, especially.

In this study, differences in adherends (occlusal or cervical) did not affect the μTBS of the adhesives. There was a report that the parallel direction of dentinal tubules to the dentin surface resulted in a higher level of bonding because of a reduction in the number of dentinal tubules and an increase in the area of intertubular dentin. The report explained that a higher amount of collagen fiber in the intertubular dentin would form sufficient hybrid layers with monomers in the adhesive system; hence, it could result in a higher level of bonding on the adherend [28]. In this study, the teeth used were cut into two segments (crown and root) at the enamel−cement junction. It might be expected that the direction of the dentinal tubule would become parallel to the adhesive surface. However, the direction of tubules observed in the SEM images of the interface was not parallel and was not so different from the occlusal surface. Furthermore, no clear difference in the number and thickness of the tubules between occlusal and cervical dentin was observed. This fact might be the reason why the difference in the adherends (occlusal or cervical) did not affect the μTBS of the two adhesives herein.

Generally speaking, hydrophobic adhesives often generate voids and/or blister like structures at dentine−adhesive interface, because dentine contains so much water (12%). The structural defects derive inferior wettability of the adhesives to a hydroscopic dentine adherend. Though the application time of 1-SEA containing novel hydrophilic amide monomer employed in the present study was shorter (10 s) than that of 2-SEA self-etching primer (20 s), no voids, blister like-structures or gap formation could be seen at the interface with SEM observation. Furthermore, interfacial failure was not observed, and mixed failure (at interface and in dentin cohesive) was dominant for both the occlusal and cervical surface. Hence, the 1-SEA containing the novel hydrophilic amide monomer employed in the present study could quickly permeate the dentin. Therefore, CUB achieved good adhesion, regardless of whether adherends were cervical or occlusal. If the new amide monomer added to CUB rapidly permeates the dentin and enamel, it might eliminate the waiting time that could reduce the risk of contamination. It might also reduce technique sensitivity and application time. Hence, fine clinical prognosis might be expected when CUB is used clinically under intraoral conditions. Future studies should focus on the clinical outcomes of the newly introduced 1-SEA containing novel hydrophilic amide monomer [29].

## 5. Conclusions

In this study, the bonding performance of a newly introduced 1-SEA containing a novel hydrophilic amide monomer was evaluated. Under the limited conditions of this study, the conclusion obtained about the novel 1-SEA was as follows:

The novel 1-SEA could quickly permeate the dentin, though the μTBS value was lower than that of the 2-SEA. As the shorter application time could reduce the risk of contamination, fine clinical prognosis might be expected when the novel 1-SEA was used clinically under intraoral conditions. On the other hand, the shorter application time could result in insufficient smear removal. So, future studies should focus on the influence of these factors: rubbing motion, application time and smear thickness, on the bonding performance of the novel 1-SEA.

## Figures and Tables

**Table 1:** Materials used in this study.

Products	Manufacturer	Code	pH	Contents
Clearfil Universal Bond Quick C (1-stepself-etch)	Kuraray Noritake Dental Co.	CUB	2.3 (adhesive)	MDP, Bis-GMA, HEMA, Hydrophilic amide monomers, Silane coupling agent, Colloidal silica, Sodium fluoride, dl-Camphorquinone, accelerator, Ethanol, Water
Clearfil MegaBond 2 C (2-stepself-etch)	-	CMB	<2.5 (primer)	Primer, MDP, HEMA, Hydrophilic dimethacrylate, Photo-initiator, Water Bond, MDP, Bis-GMA, HEMA, Hydrophilic dimethacrylate, Photo-Inisiator, Silaneted colloidal silica
Clearfil AP-X (composite resin)	-	-	-	Bis-GMA, TEGDMA, Silaneted Barium glass filer, Silaneted colloidal silica, dl-Camphorquinone

MDP: 10-Methacryloxydecyl dihydrogen phosphate. Bis-GMA: 2-bisphenol A diglycidyl methacrylate. HEMA: 2-hydroxyethyl methacrylate. TEGDMA: Tryethyleneglycol dimethacrylate.
